# Broad Reactivity Single Domain Antibodies against Influenza Virus and Their Applications to Vaccine Potency Testing and Immunotherapy

**DOI:** 10.3390/biom11030407

**Published:** 2021-03-10

**Authors:** Andrew Tung Yep, Yasu Takeuchi, Othmar G. Engelhardt, Simon E. Hufton

**Affiliations:** 1 Biotherapeutics Division, National Institute for Biological Standards and Control (NIBSC), Potters Bar, Hertfordshire EN6 3QG, UK; andrew.tungyep@nibsc.org; 2 Division of Infection and Immunity, University College London, London WC1E 6BT, UK; y.takeuchi@ucl.ac.uk; 3 Advanced Therapies Division, NIBSC, Potters Bar, Hertfordshire EN6 3QG, UK; 4 Division of Virology, NIBSC, Potters Bar, Hertfordshire EN6 3QG, UK; othmar.engelhardt@nibsc.org

**Keywords:** single domain antibodies, nanobodies, influenza, immunotherapy, vaccine potency testing

## Abstract

The antigenic variability of influenza presents many challenges to the development of vaccines and immunotherapeutics. However, it is apparent that there are epitopes on the virus that have evolved to remain largely constant due to their functional importance. These more conserved regions are often hidden and difficult to access by the human immune system but recent efforts have shown that these may be the Achilles heel of the virus through development and delivery of appropriate biological drugs. Amongst these, single domain antibodies (sdAbs) are equipped to target these vulnerabilities of the influenza virus due to their preference for concave epitopes on protein surfaces, their small size, flexible reformatting and high stability. Single domain antibodies are well placed to provide a new generation of robust analytical reagents and therapeutics to support the constant efforts to keep influenza in check.

## 1. Introduction

Influenza A and influenza B viruses circulate within the human population and give rise to seasonal epidemics which are kept in check with an annual vaccination program. Influenza A represents a further public health challenge as it can cross the species barrier between animals and humans leading to occasional pandemics with potentially very high mortality and morbidity. Although vaccines are the main method of infection control, their timely implementation presents significant challenges [1,2,3,4]. These include the predictions of which strains will emerge and be most prevalent in the population, extended lead times for vaccine production and poor immunogenicity in certain vulnerable patient groups [3]. The concept of a ‘universal therapy’ which overcomes the virus’s ability to evade the immune system through constantly changing the structure of its viral coat is a long-standing objective of those developing novel therapeutic interventions for influenza [5,6,7,8,9,10]. Monoclonal antibodies provide one such possible route to a universal therapy. For several decades, it was believed that antibodies to influenza A virus would only bind a single strain or a narrow range of viral strains. However, there are individuals who have immunity to strains to which they have not previously been exposed, which suggested that cross protective immunity is indeed possible and that epitopes that can elicit such a response exist [11,12]. This was confirmed in 1993 with the isolation of a monoclonal antibody, C179, that could neutralize viruses belonging to several different subtypes [13]. This antibody, unlike previously characterized neutralizing influenza antibodies, tested negative for hemagglutination inhibition. The hemagglutination inhibition assay (HAI) was the main assay up to that point for evaluation of viral neutralization and tests the interaction of viral HA with its sialic acid receptor after exposure to antibody. A negative result indicates that the interaction is unaffected whereas a positive result indicates that the antibody is binding at or near to the sialic acid receptor binding site in the HA head region [14]. The authors correctly hypothesized that the antibody bound away from the sialic receptor binding site and in the hemagglutinin stem region, which has a higher level of conservation than the head region, explaining the cross-reactivity [15]. It was only when several other stem binding broadly neutralizing antibodies were reported in 2008 that the significance of the discovery of C179 became clear [16,17,18].

The isolation of cross-neutralizing monoclonal antibodies to the more conserved hemagglutinin (HA) stem region [16,17,18,19,20] was a surprising finding and raises the question why it has been so difficult to identify this type of antibody in the past. The answer may, at least partially, lie in the virus adapting to reduce the immunogenicity of its most important and conserved determinants of function, combined with the challenges human antibodies have in accessing these parts of the viral coat structure. Antibodies to these conserved epitopes are likely to be rare and it is only through the advent of sophisticated antibody engineering techniques such as B cell cloning [21] and phage display [22,23] that it has become easier to isolate such monoclonal antibodies. Two of the first examples are the human monoclonal antibodies (mAbs) F10 [18] and CR6261 [17] which have both been shown to bind to a highly conserved pocket in the membrane proximal stem region. In addition, these mAbs were shown to use only their heavy chain for antigen binding, with no contact being made by their respective light chains. This unusual property has also been reported for several subsequent cross-neutralizing antibodies such as C05, F045-092 and 27F3 which have also demonstrated ‘heavy chain only’ binding including to regions other than the HA stem [24,25,26]. This could suggest that ‘heavy chain only’ recognition may be an optimal mode of binding for broadly neutralizing antibodies to influenza as has been proposed for cross-neutralizing antibodies to HIV [27,28]. These observations imply that the VL domain may not be required in accessing these important viral epitopes and furthermore could be a hindrance. The existence of naturally occurring ‘heavy chain only’ antibodies is well documented in camelid species [29,30] and sharks [31,32] and their unique properties are being exploited for wide ranging applications in biotechnology including immunotherapy [33].

Similar to conventional VH domains, camelid VHH domains are comprised of four framework regions (FR1-4) and three complementary determining regions (CDR1-3). However, they have several key differences in the FR2 and the CDRs which compensate for the absence of a paired light chain. Four highly conserved substitutions in the FR2 create a hydrophilic exposed patch at the surface which would normally interact with the VL domain in a conventional antibody. In addition, the CDR3 loop often folds back over this exposed interface which would sit between the VH and VL domains in conventional antibodies. It is also well documented that the CDR3 length of VHH domains is often but not always longer than in conventional antibodies and, to compensate for the associated extra flexibility, it can form a disulfide bond with the CDR1, CDR2 or FR2 [34,35,36]. Single domain antibodies (sdAbs) can be isolated from immunized, naïve or synthetic libraries [37,38,39] constructed using V genes from camelids [29] or from human donors [40,41,42]. The sdAb format has several advantages over conventional IgG based monoclonal antibody formats which comprise both a light chain and heavy chain. These advantages include (i) small size (~15kDa), (ii) low cost microbiological production, (iii) flexible formatting, (iv) high stability and (iv) potential to access concave or hidden epitopes [33,43,44,45,46,47,48]. This is particularly pertinent to targeting viral proteins where epitopes are often hidden by deep invaginations or canyons and may be difficult to access effectively by conventional monoclonal antibodies which have a preference for binding to larger flat surfaces [49,50,51,52]. Furthermore, it has been observed that human monoclonal antibodies to influenza HA show biases for certain germline segments and can form functional broadly neutralizing antibodies with these segments after very limited somatic hypermutation in vivo. This sequence convergence suggests that such antibodies are the result of an early or immediate response to infection potentially guided by previous exposure to influenza [53,54,55]. Alternative approaches to generating mAbs which have been extensively optimized in vivo, such as through immunization of camelids, could be expected to yield molecules of higher affinity and with different binding properties to those generated by the human immune system.

This review summarizes the status and applications of single domain antibodies with broad reactivity against influenza virus. We review their different mechanisms of action and their breadth of reactivity. In addition, we discuss how targeting conserved epitopes, reformatting to multi-domain molecules or generating oligoclonal cocktails of sdAbs may provide complementary routes to mitigate the virus’s ability to change and escape detection for applications in vaccine potency testing and as a new class of immunotherapeutics.

## 2. Conserved Targets on Influenza Virus and Mechanisms of Action of Single Domain Antibodies

**Figure 1:**
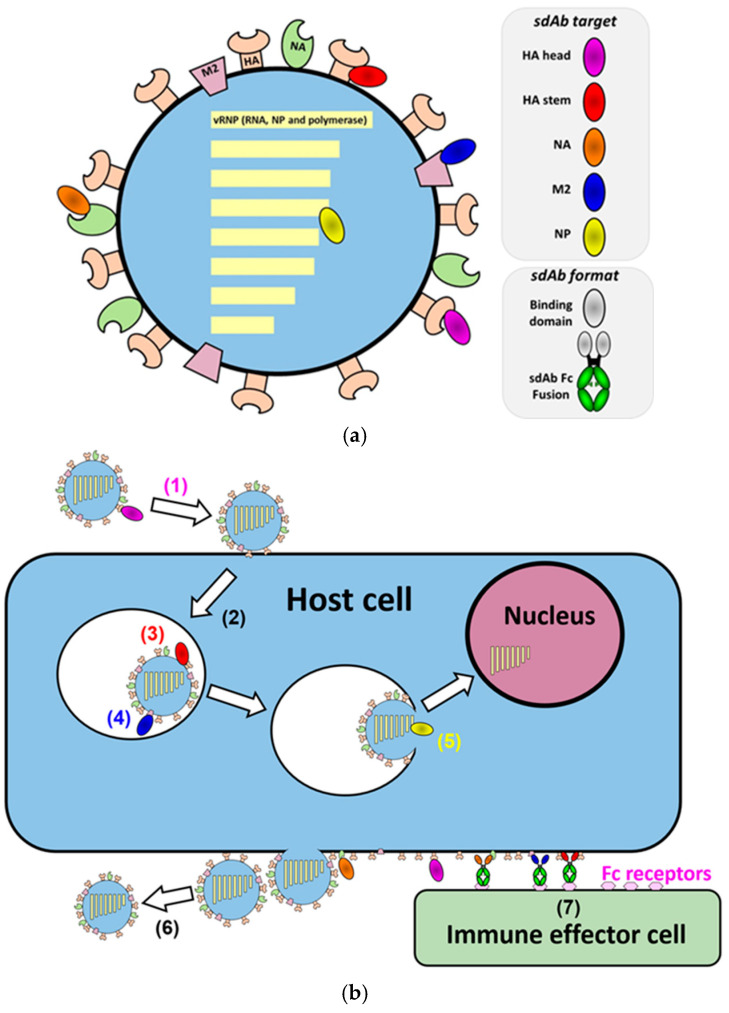
Relationship between sites of antibody binding and mechanisms of inhibition of infection of influenza virus. (**a**) Cartoon of influenza virus, its envelope proteins, the sites targeted by anti-influenza sdAbs and different sdAb formats. HA head (sdAb binding to hemagglutinin head domain), HA stem (sdAb binding to hemagglutinin stem domain), NA (sdAb binding to neuram-inidase), M2 (sdAb binding to M2 ion channel), NP (sdAb binding to nucleoprotein, a component of the vRNP complexes) (**b**) Cartoon depiction of parts of the influenza virus infection cycle and the various methods by which antibodies inhibit the spread of the virus depending on their epitope. The infected host cell, influenza virus, nucleus and effector cell are indicated (1) Virus attachment to host cell inhibited by HA head antibodies. (2) Endocytosis of virion. (3) pH mediated fusion of virus and host membranes inhibited by HA stem antibodies. (4) pH mediated proton influx inhibited by some M2 antibodies interfering with uncoating. (5) Transport of ribonucleoproteins to the nucleus inhibited by NP antibodies. (6) Inhibition of dissociation of new virions from host cells by anti-NA antibodies. (7) Immune effector functions such as antibody dependent cellular cytotoxicity (ADCC) mediated by Fc-linked HA stem, NA and M2 antibodies.

**Figure 2:**
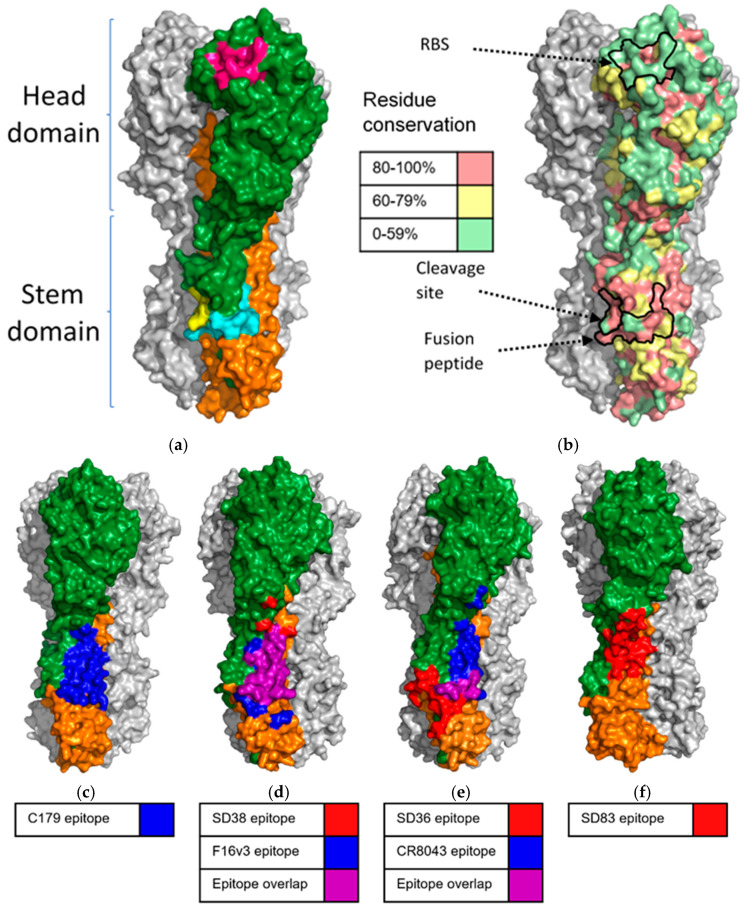
Structure of HA and epitope footprints of HA stem binding sdAbs and conventional antibodies with similar ranges of cross-reactivity. (**a**) Structure of hemagglutinin (A/California/04/2009, PDB code 3LZG) [84]. One unit of the trimer is highlighted, HA1 is shown in green and HA2 is shown in orange. The fusion peptide, which, upon pH mediated conformational change, straightens and inserts into the host membrane is highlighted in cyan. The residues immediately surrounding the HA0 cleavage site are highlighted in yellow. The receptor binding site is shown in pink. (**b**) The same hemagglutinin structure is used to display a heat map of influenza residue conservation across 6 influenza A subtypes known to infect humans (H1, H2, H3, H5, H7 and H9) [85]. Key residues in the RBS, fusion peptide and cleavage site are highly conserved as well as many residues in the stem region at the HA1/HA2 interface. (**c**–**f**) Hemagglutinin trimer structures are used to display binding sites of sdAbs (blue), conventional antibodies (red) and areas of epitope overlap (purple). (**c**) The original stem binding antibody C179 is displayed on A/California/04/2009. (**d**) SD38 and F16v3 epitopes displayed on A/Hong Kong/1/68 HA (PDB code 4FNK) [24]. (**e**) SD36 and CR8043 epitopes displayed on A/Hong Kong/1/68 HA. SD38 and pan influenza A conventional antibodies have a high degree of overlap in their epitope footprints while SD36 and Group 2 stem binding conventional antibodies have sharply contrasting epitope footprints. This may indicate a difference in binding preference between conventional antibodies and sdAbs. (**f**) SD83 epitope displayed on B/Brisbane/60/08 (PDB code 4FQM) [70]. The SD83 epitope has a greater degree of contact with HA2 compared to SD36 and SD38. The residues included in the epitope footprint were determined based on protein structures in complex (PDB codes 6FYU, 6FYT, 6FYW, 3ZTJ, 4NM8) [69,74,86] using the PBDePISA online tool [87].

It has been proposed that cross-protective immunity seen in some individuals is due to immune responses to highly conserved structures on the virus [11,12]. These include regions of the viral coat protein hemagglutinin (HA) [17,18], the extracellular domain of the M2 ion channel [56,57], neuraminidase (NA) [58,59] and the internal nucleoprotein (NP) [60]. Hemagglutinin is the main viral coat protein and is responsible for viral entry through binding to sialic acid on the surface of cells [61] (Figure 1a). Sequence analysis reveals that there is considerable variation in the HA gene which can be classified into 18 different subtypes of two phylogenetically distinct groups, group 1 (H1, H2, H5, H6, H8, H9, H11, H12, H13, H16, H17 and H18 subtypes) and group 2 (H3, H4, H7, H10, H14 and H15 subtypes), which in combination with eleven different neuraminidase subtypes (N1 to N11) describes all known influenza A viruses [62,63]. Each of these two phylogenetic groups has a highly variable globular head region consisting of the HA1 subunit which mediates sialic acid receptor binding and a more conserved proximal stem region which is principally comprised of the HA2 domain and some of the HA1 domain [61] (Figure 2a,b). Similarly, influenza B viruses co-circulate in the human population as two antigenically distinct lineages called the Victoria lineage and the Yamagata lineage [64,65,66].

All three viral envelope proteins, HA, NA and M2 as well as the internal structural protein NP are promising targets for antibody-based viral therapy and analytical testing. The structures of these viral proteins show many deep crevices and canyons on the surface which can present a challenge of accessibility for conventional monoclonal antibodies which have a preference for targeting larger flatter epitopes [44,49,50,52]. These concave protein regions may be better targeted by sdAbs [44,48]. The mechanisms by which sdAbs can inhibit the influenza virus are intimately linked with the function of the target protein and their corresponding epitope (Figure 1b, Table 1) [67]. To date, most scientific effort has focused on conventional monoclonal antibodies from human donors, which has revealed many of the vulnerabilities of influenza virus and associated epitopes, with less known about how sdAbs mediate virus neutralization. Nevertheless, it is clear that there is substantial similarity between inhibitory mechanisms of these two types of antibodies. However, as the list of sdAbs against influenza grows, subtle differences related to the structure and function of sdAbs can be expected to be revealed.

### 2.1. Single Domain Antibodies against Influenza Hemagglutinin

As the major envelope protein of the influenza virus, HA has by far attracted the most attention as a target for sdAbs. HA is assembled by host cells as a homotrimer with each monomer comprising two subunits, HA1 and HA2 (the precursor is referred to as HA0) [61]. Upon budding of the virion from the host cell, the HA0 monomers are cleaved by host proteases, completing the maturation of the virion. The receptor binding site (RBS) sits within the globular HA1 head domain and binds sialic acid on prospective host cell membranes resulting in endocytosis of the virus particle (Figure 1b and Figure 2a). The drop in pH in the host cell endosome causes conformational change in the stem region of the molecule mediated by HA2 and results in the insertion of a hydrophobic fusion peptide into the endosome membrane. This insertion catalyzes the fusion of viral envelope and endosomal membrane, releasing the contents of the virion into the host cell (Figure 1b) [61,68].

The first cross-neutralizing influenza antibody discovered, C179, binds to a highly conserved site on the stem of HA which mostly consists of residues from HA2 but also makes some contact with the stem portion of HA1 (Figure 2c) [15]. The high level of conservation between influenza viruses of many of the residues in the HA stem allows antibodies with epitopes overlapping and adjacent to the C179 epitope to have exceptionally broad cross-reactivity. Several have been discovered with pan-influenza A reactivity [20,69] and even cross-reactivity that can be extended to influenza B viruses [70]. The most conserved residues of the stem region are those which catalyze the large low pH induced conformational changes in the stem including residues in the fusion peptide which inserts into the cell membrane (Figure 2a,b). The function of these residues in the HA stem region is absolutely conserved between all influenza A and B viruses [61,71] and human monoclonal antibodies binding to this region have been shown to block these low pH induced conformational changes, thereby inhibiting membrane fusion [17,18,70,71] (Figure 1b).

There is significant evidence that cross-reactive stem-binding sdAbs can neutralize influenza viruses by similar mechanisms of inhibiting the pH-induced conformational changes in the HA stem. R1a-B6, an sdAb isolated from an immunized alpaca phage display library, has been described with broad group 1 cross neutralizing activity and a characteristic absence of hemagglutination inhibition [72]. This placed this sdAb in the same category as the seminal group 1 cross-subtype neutralizing human mAbs CR6261 [16,17] and F10 [18]. Furthermore, it was demonstrated that R1a-B6 binding to HA was lost after low pH treatment suggesting that its epitope sits in the HA stem region. Subsequently, epitope mapping of R1a-B6 using yeast display mutational scanning identified residues in the stem region overlapping the fusion peptide which, when mutated, specifically disrupted R1a-B6 binding [73]. In a more recent report, three broadly neutralizing sdAbs (SD38, SD36, SD83) against influenza HA were shown through their crystal structures to bind to highly conserved epitopes in the HA stem region and the low-pH induced conformational change could be inhibited by their binding [74] (Figure 1b and Figure 2, Table 1).

Cross-reactive sdAbs have been isolated which, although binding to the HA stem, behave differently to other stem-binding sdAbs and show increased binding at low pH. One such sdAb, R2b-D8 showed broad group 1 cross-subtype reactivity and mapped to a peptide epitope in the stem region but was not able to neutralize virus in vitro [72]. Similarly, sdAbs with group 2 cross-reactivity, which includes H3N2 and H7N9 subtypes, could be mapped to conserved epitopes in the HA stem region using yeast display and mutational scanning [75]. These sdAbs also showed enhanced binding at low pH, which was consistent with their binding to a HA stem epitope which was either partially or entirely obscured at neutral pH. None of this group of sdAbs have been characterized in animal challenge models, so any potential for inhibiting viral infection is unknown. However, there may be applications in assessing the conformational integrity of HA in vaccine samples (Section 6).

Due to its vital interaction with sialic acid, the receptor binding site (RBS) is also a highly conserved site on HA (Figure 2a,b). However, unlike the HA stem region there are a limited number of highly or absolutely conserved residues within this site. The head region of HA is the prime target of the immune system [76], which drives constant antigenic change in this region [77], especially around the periphery of the RBS [78,79]. As such, targeting the head domain presents a higher hurdle for antibodies to achieve broad cross-reactivity. Frequently, RBS-binding conventional antibodies make some contacts to more variable residues [67] and generally have a narrower reactivity range within a subtype compared to stem binders [67,80]. Single domain antibodies have been described which bind to epitopes in the HA1 head domain of H1N1, H5N1, H3N2, H7N9 and influenza B-HA. Similarly, they have a generally narrower range of reactivity limited to within a subtype compared to those targeting the HA stem [72,73,74,81,82,83]. To date only one structure of a head binding sdAb (SD84) in complex with HA has been described specific for influenza B-HA [74] and it was shown to bind directly to the RBS and to neutralize influenza B virus through blocking virus attachment to cells.

### 2.2. Single Domain Antibodies against Influenza Neuraminidase

Neuraminidase (NA), although less abundant than HA, nevertheless plays a crucial role in the infection process and is required for the dissociation of a newly formed virion from the surface of an infected cell. NA exists on the cell surface as a homotetramer with one enzymatic site per monomer. The active site cleaves sialic acid (SA) bound to neighboring HA molecules, so releasing virus from the cell surface (Figure 1b). HA will inevitably bind to SA on the surface of the infected cell while a new virion is budding so the enzymatic activity of NA prevents virions from accumulating on the surface of infected cells. Virion accumulation is observed when NA is inhibited by drugs or antibodies [88]. The prospect of inhibiting the function of NA makes it a good target for sdAbs which have well documented propensity to bind to enzyme active sites [44]. Single domain antibodies against NA have been isolated and have demonstrated inhibition of neuraminidase activity and in vivo efficacy in a mouse challenge model (Figure 1b, Table 1) [89]. In another study a llama was immunized with multiple NA subtypes and sdAbs were selected based on binding to each subtype. Many of the sdAbs selected possessed some degree of subtype cross-reactivity including one sdAb which bound to 8 of the 9 subtypes tested. The majority of sdAbs tested showed neuraminidase inhibition activity, which suggests that they bound to conserved epitopes at or around the active site [90].

### 2.3. Single Domain Antibodies against Influenza M2 Pore

M2 is a homo-tetrameric single pass ion pore. Like HA, it is activated by the acidification of the endosomal environment after the virus is endocytosed, becoming permeable to protons which then diffuse into the virion. The drop in pH initiates the genome unpacking process in preparation for release of virion contents into the cytoplasm [91,92]. The external domain of M2, referred to as M2e, consists of only 23 amino acids, all of which are highly conserved [93]. Passive transfer of conventional human monoclonal antibodies to M2e have been shown to be protective in animal models [56,57]. However, these antibodies do not appear to mediate their effect by direct functional inhibition of the M2 pore and the infection process. Instead, they mediate protection through the elimination of infected cells by Fc-mediated effector functions such as ADCC (antibody dependent cellular cytotoxicity) and CDC (complement dependent cytotoxicity), not by viral neutralization. By contrast, a single domain antibody against M2e, M2-7A, has been shown to neutralize virus and protect mice from lethal challenge in its monovalent format, without any engagement of Fc effector functions. M2-7A appears to block the function of the pH activated proton channel, interfering with the essential virus uncoating process (Figure 1b, Table 1) [39]. In more recent studies, bispecific sdAbs have been described which can engage with both M2e and also FcγRVI so as to recruit macrophages to virus infected cells. This novel M2e sdAb molecule was shown to have protective efficacy in a mouse challenge model when delivered either intranasally as purified protein [94] or as encoding mRNA for sdAb production in situ within the lungs [95].

### 2.4. Single Domain Antibodies against Influenza Nucleoprotein

The viral nucleoprotein (NP) is a highly conserved protein essential for nuclear trafficking and packaging of the influenza virus genome. It is internal to the virus unlike the other conserved viral protein targets, making it a uniquely interesting target for intervention [60]. Single domain antibodies against NP have been described which are capable of disrupting virus replication by preventing nuclear import of viral ribonucleoproteins (vRNPs) (Figure 1b, Table 1) [96,97]. Schmidt et al. have described an efficient lentiviral screening system whereby intracellularly expressed sdAbs that confer a cellular phenotype can be identified. Using this system, a panel of sdAbs against NP was identified which was capable of protecting human cells from viral infection by preventing nuclear import of vRNPs [98]. Although representing a unique mechanism of action, the therapeutic application of NP binding sdAbs would require intracellular delivery of the sdAb either as recombinant protein or as the encoding genetic material to a sufficiently high enough proportion of infected cells to inhibit an influenza infection [99,100].

**Table 1:** Single domain antibodies against influenza and their mechanism of action.

Site of Binding	Examples sdAbs	Reactivity	Epitope	Mechanism of Action	Reference
**HA (head region)**	H5-VHHm	H5N1	K189 ^(a)^	Direct occlusion of sialic acid binding site inhibiting host cell attachment.	[83]
	aHA-7	H5N2	Not determined (ND)		[82]
	SD84	IBV ^(^^d)^	Pdb: 6CNW ^(b)^		[74]
	NB7-14	H7N9	D67, S135 ^(a)^		[75]
	R1a-G6	H1N1	I169, D171, G173 ^(a)^		[72]
	NB3-02	H3N2	F193, Y195 ^(a)^		[75]
	Vic 2a-6	Victoria IVB	H122, N129 ^(a)^		[81]
**HA (stem region)**	SD38	H1, H2, H5	Pdb: 6FYT ^(b)^	Binds to fusion machinery, inhibiting fusion of virus and endosome membranes.	[74]
	SD36	H3, H4, H7, H10	Pdb: 6FYU ^(b)^		[74]
	SD83	IBV	Pdb; 6FYW ^(b)^		[74]
	R1a-B6	H1, H5, H2, H9	G20, W21, I45 ^(a)(c)^		[72]
	Vic 1b-10	IBV	ND		[81]
**NA**	N1-VHHb	H5N1	I437T ^(b)^	Enzyme inhibition preventing budding virions from leaving host cells.	[89]
**M2**	M2-7A	H1, H3	SLLTEVET epitope	Inhibition of M2 proton channel, interfering with viral uncoating.	[39]
	M2e-VHH-23m	H3N2	PDB 6SOY		[94]
**NP**	αNP-VHH1		Pdb: 5TJW ^(b)^	Binding to and blocking nuclear import of viral ribonucleoproteins.	[96]

^(a)^ Refers to mutations identified that inhibit binding either using viral escape mutagenesis or yeast display mutational scanning ^(b)^ Refers to the pdb accession number of the crystal structure ^(c)^ Numbering starts in the HA2 region ^(d)^ IBV is influenza B virus, HA—Hemagglutinin, NA—Neuraminidase, M2—Matrix pore 2, NP—Nucleoprotein.

## 3. Epitope Footprint, Contact Residues and Reactivity Range of Single Domain Antibodies against Influenza Hemagglutinin

The range of cross-reactivity of antibodies to influenza virus varies considerably and is inevitably defined in terms of specificity to historical influenza strains. All cross-reactive influenza antibodies rely on a binding site with a high level of sequence conservation between strains. However, within these sites certain residues will be almost absolutely conserved while others will vary substantially depending on the virus strain (Figure 2b) [67]. Binding interactions dependent only on very highly conserved residues will be preserved across many subtypes, whereas binding which is dependent on more variable residues will ultimately lead to a narrowing of reactivity range [6]. In assessing the reactivity range of mAbs the specificity is tested against pre-existing strains going back in time with the expectation that demonstration of broad reactivity will be preserved if the epitope does not change as the virus evolves. Techniques that can predict if an antibody will retain binding to an emerging virus may prove useful in future outbreaks and virus monitoring. For example, mutational escape using yeast display when coupled to next generation sequencing has been described and shows promise in predicting which mutations have the potential to affect antibody binding and which do not so that, when a new strain emerges, the impact on binding can be quickly assessed. Recent studies have described sdAbs with specificities which can be correlated to natural sequence differences through the generation and testing in parallel large repertoires of mutations using yeast display mutational scanning (Table 1) [73,75,81]. Single domain antibodies with either cross-reactive or lineage specific binding to influenza B virus (IBV) strains included in seasonal vaccines spanning over 20 years have been described. Furthermore, lineage specific binding could be mapped to an epitope comprising natural sequence divergence between Victoria and Yamagata IBV lineages at residue 122 in the HA1 domain (Table 1) [81]. Lineage specific sdAbs against IBV may have applications for vaccine potency assays where quantitation of HA from both IBV lineage strains included in seasonal vaccines is required [101]. Subtype specific sdAbs against H7 influenza have been described and their binding has been mapped to distinct epitopes in the HA1 domain which correlated with their breadth of reactivity. For example, NB7-14 was mapped to a highly conserved epitope in the HA1 domain with specific residues conserved across all H7N9 strains from its emergence in human populations in 2013 up to 2017. Lineage specific sdAbs against H7N9 have also been described which are capable of distinguishing low pathogenic from high pathogenic H7N9 viral strains which could be correlated with natural sequence divergence using yeast display mutational scanning [75].

Evolving escape mutations to an antibody is made harder for a virus if these mutations cause a significant fitness cost to the virus or very few mutations have the ability to facilitate viral escape. It follows therefore, that antibodies targeting smaller epitopes containing fewer residues in highly functionally conserved regions would be the most resistant to escape. One of the earliest examples which lends weight to this concept is the unusual fully human antibody C05. This conventional monoclonal antibody mediates cross-neutralization of influenza A virus through a very long single CDR3 loop which penetrates the sialic acid binding pocket, forming key interactions with highly conserved residues whilst avoiding the more variable HA loops surrounding the RBS [24]. This antibody can be seen as analogous to a single domain antibody as all the binding function is largely limited to its CDR3 loop to the point where it can be removed from the immunoglobulin fold and retain activity as a constrained peptide. In addition, some broadening of cross-reactivity can be achieved through removing contacts made by the CDR1 with more variable residues around the RBS [102]. This demonstrates that it is possible to enhance the breadth of cross-reactivity through minimizing epitope contacts outside of conserved epitope residues either through design or selection. An analysis of the paratopes of sdAbs to a wide range of pathogens and human proteins in comparison with conventional antibodies revealed that paratope area, and therefore epitope area, is on average smaller than that of conventional antibodies [103]. However, thus far, for sdAbs against influenza this does not appear to have translated into a clear advantage in breadth of reactivity [45,103].

To date there are three available crystal structures for stem binding sdAbs in complex with HAs which present an opportunity to compare their epitope footprints to each other and to functionally equivalent conventional stem binding antibodies (Figure 2d–f). The sdAbs SD36, SD38 and SD83 cross-neutralize influenza A group 2, influenza A groups 1 and 2 and influenza B, respectively [73]. Despite their contrasting reactivity ranges, the antibodies have significantly overlapping epitopes with several homologous residues forming part of the footprint of all three antibodies. SD36 and SD38 have the most similar footprints with both epitopes sitting within the conserved hydrophobic groove at the junction between HA1 and HA2. Meanwhile the epitope footprint of SD83 is slightly higher on the stem and includes more of HA1 than either SD36 or SD38 [74].

Conventional antibodies with group 1 and 2 cross-reactivity such as F16, 27F3 and MEDI8852 and multiple others have been discovered and visualized in complex with HA. The epitope footprints of these antibodies are remarkably convergent with a core of several residues high up on the stem near the HA1/HA2 interface contacted by almost every antibody in this group. Despite the difference in antibody type, SD38 fits neatly into this class of antibody in both epitope footprint and reactivity range (Figure 2d) [25,69,104,105].

The footprint of SD36 on the other hand, differs significantly from functionally equivalent influenza A group 2 stem-binding conventional antibodies. The epitopes of CR8020, CR8043 and 9H10 overlap with each other and bind further down the stem than SD36 [86,106]. The three conventional antibodies make the most of their vital contacts to the highly conserved fusion peptide so in addition to inhibiting membrane fusion, they are also able to inhibit HA0 cleavage and maturation, thus preventing influenza virus from becoming infectious. By contrast, SD36 has an epitope footprint more typical of group 1 and 2 cross-neutralizing antibodies, forming contacts with the same core group of residues as these antibodies and lacking the ability to block HA0 maturation [107]. More general differences in the binding preferences of conventional antibodies and sdAbs may contribute towards these observations but further research and characterization of more sdAbs is needed to draw any firm conclusions (Figure 2d,e).

## 4. Reformatting and Optimization of sdAbs for Different Applications in Targeting Influenza

**Figure 3:**
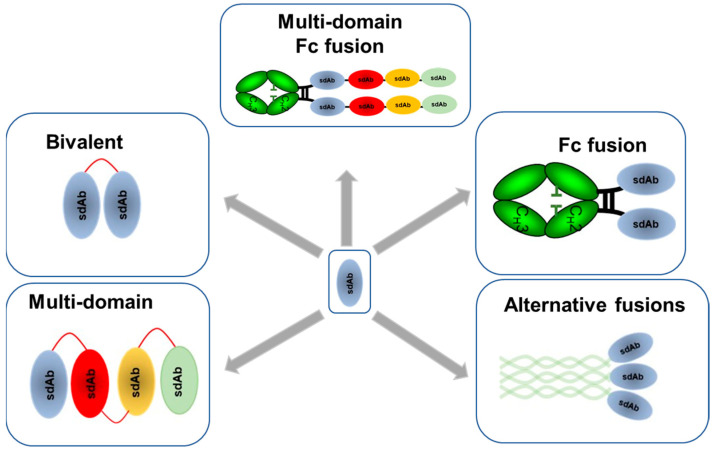
Single domain antibody formats used to optimize functional targeting of influenza virus. sdAb specific to different targets on influenza can be optimized through re-formatting: bivalent format to enhance potency [72,75,83,90,108]; multi-domain fusions to enhance potency and breadth of reactivity [74]; multi-domain Fc fusions to enhance potency, breadth of reactivity, extent half-life to reduce dosing and incorporate effector function [74]; Fc fusion to enhance potency, extend half-life to reduce dosing and incorporate effector function [74,90,109]; alternative fusion partners to enhance potency and extend half-life to reduce dosing [82,110].

While the binding interaction between influenza virus and antibody is the primary determinant of the mechanism of action and the breadth of reactivity, another vital aspect to function is determined by the antibody format. Single domain antibodies, because of their simple structure and single open reading frame, can easily be re-designed to enhance function and potency in line with different applications and delivery methods. This can be by increasing avidity, engaging effector functions, enhancing breadth of reactivity, mitigating viral escape, enhancing pharmacokinetics or enhancing stability (Figure 3).

### 4.1. Multivalent and Multi-Paratopic sdAbs

Although single domain antibodies can have potent binding and neutralization activity on their own, their activity can be radically enhanced by multimerization to introduce avidity. Single domain antibodies can be made bivalent by fusing them with an Fc region [74,89,109], by fusing two identical sdAb domains together using a flexible peptide linker [72,75,82,108] or by fusion to a dimerization domain such as a leucine zipper [82]. The relationship between avidity and potency is related to the mechanism of action. Single domain antibodies which neutralize influenza through blocking virus attachment to cells demonstrate substantial increases in potency in a bivalent format [26,72,75,108]. However, for sdAbs which target the HA stem the relationship between avidity and potency is less clear [72,111]. Early studies with the seminal broadly neutralizing stem binding human antibody CR6261 showed that both the monovalent Fab and the bivalent IgG had similar neutralization potencies suggesting that bivalency was not essential or beneficial for antibodies which neutralize influenza virus post-viral attachment [17]. Subsequent studies with an equivalent sdAb, R1a-B6, showed similar results in that bivalency did not increase maximum levels of neutralization activity of H1 and H5 subtypes. However, somewhat surprisingly, the breadth of cross-subtype neutralization activity was seen to increase to include more divergent influenza subtypes H2 and H9 [72]. The authors speculated that this may be due to the cellular uptake of the influenza virus being a rate-limiting step in the efficacy of R1a-B6 which neutralizes the virus post-viral attachment, suggesting there may be a ceiling above which the potency cannot be enhanced by avidity for stem-binding sdAbs. However, avidity is suggested to enhance lower affinity interactions with more divergent subtypes so increasing the breadth of neutralization reactivity [72].

Although an important addition to the treatment options for influenza, the widespread use of antiviral drugs like oseltamivir and rimantadine will inevitably lead to the development of resistance and lower efficacy over time [112]. The same selection of resistant strains could also be a consequence of future widespread use of antiviral mAbs against influenza even when targeting highly conserved epitopes such as those in the HA stem [113]. Additional approaches to mitigate this risk are required and strategies to neutralize virus through two or more distinct epitopes with non-competing antibodies are expected to be required. The principle behind this is the notion that influenza virus is less likely to generate mutations at two distinct epitopes than one. This can be achieved through the use of mixtures of antibodies against different epitopes [114] or through the design of multi-domain sdAbs capable of interacting with several distinct epitopes on the influenza virus simultaneously [74]. This potential has been highlighted by MD3606 which has four pairs of different broadly neutralizing sdAbs directed towards influenza A and B and demonstrates nearly universal neutralization of all influenza A and B viruses. The sdAb domains SD83 and SD84 bind to the HA stem and RBS of influenza B viruses, respectively, providing an extra level of protection from influenzas B virus escape. Although MD3606 utilizes a multi-domain format for broadening reactivity to diverse influenza subtypes the concept can equally be applied to target non-overlapping epitopes on the same target antigen so mitigating viral escape [74].

### 4.2. Incorporation of Immune Effector Functionality into sdAbs 

Conventional monoclonal antibodies binding to HA stem, NA and M2e have all been shown to benefit from Fc-mediated neutralization of influenza in addition to their direct neutralization mechanisms [56,70,88]. As sdAbs are not naturally fused to an human Fc region, they require this function to be added so antigen recognition can functionally engage with the host immune system through Fcγ receptors [115,116]. This can enhance the potency of sdAbs by adding further mechanisms of neutralization mediated by immune effector cells [115]. The Fc region, when bound to an influenza infected cell, is able to recruit effector cell types such as macrophages and natural killer cells and mediate antibody dependent cellular cytotoxicity (ADCC), complement dependent cytotoxicity (CDC) or antibody dependent cellular phagocytosis (ADCP) [117]. In cases where sdAbs have insufficient direct neutralization potency then the role of the Fc function is more important. This has been demonstrated with a sdAb against NA formatted as an Fc fusion which despite not having any anti-viral activity in vitro was able to protect mice from a lethal challenge with H5N1 virus [89]. The additional potency possible through engaging Fc effector function has been shown with the multivalent sdAb MD3606 where Fc variants of MD3606, with binding to Fcγ receptors disabled, showed a significant decrease in protective efficacy relative to a variant with functional Fcγ receptor binding [74]. More recent studies with the broadly neutralizing sdAb R1a-B6 against the HA stem showed less of a reliance on effector function. R1a-B6 was shown to have equal protective efficacy when formatted as an Fc fusion with a mouse IgG2a or as a mouse IgG1 which has no ADCC activity [109].

### 4.3. Extending Half-Life of sdAbs

Single domain antibodies have a molecular weight of around 15kDa and a half-life of approximately 90 min in vivo [118,119] as the kidneys swiftly remove any molecules with a molecular weight of less than 30-50kDa. For any therapeutic or prophylactic application, antibodies with such a short half-life are unable to sustain sufficiently high concentrations in the systemic circulation to inhibit influenza virus. Further functionality is required to improve their pharmacokinetics. Alternatively, modifications of their delivery method can help maintain an effective concentration of antibody in vivo [120]. Several strategies have been described for improving the pharmacokinetics of sdAbs against influenza through modification to reduce renal clearance or through gene therapy [99].

Conceptually, the simplest approach to increasing the half-life of sdAbs is to increase their hydrodynamic size and therefore reducing filtration through the kidneys. This can be achieved by linking several sdAbs together by means of flexible linkers [72,83,89,108]. If a sufficient number of sdAbs can be fused together to limit filtration through the kidneys, then additional half-life enhancing technologies are not required. This approach may have an upper limit and to date the maximum number of sdAbs that have been fused together head-to-tail is four [74]. Other approaches to increase size have been described which involve engineering ferritin to display a sdAb (nanobody) which then self-assembles into a large complex called a ‘fenobody’ [110]. Using this approach, the authors were able to demonstrate a tenfold increase in half-life in a mouse model and a 360-fold higher affinity against H5N1 virus. Chemical conjugation to polyethylene glycol (PEG) or to negatively charged polymers such as hydroxyethal starch (HESylation) have been used in a number of licensed antibody products, however to date no PEG modified sdAb against influenza has been described and tested for in vivo efficacy [121]. 

A frequently used approach to extend the half-life of sdAbs is to fuse them directly to an antibody Fc region. This allows sdAb Fc fusions to achieve a comparable half-life to conventional monoclonal antibodies by utilizing the normal mechanism of FcRn recycling through the kidneys [74,109,122]. Fusion of sdAbs to an Fc region also benefits from linkage to the effector arm of the immune system so potentially enhancing potency. Alternatively, therapeutic sdAbs can be fused to sdAbs specific for long-lasting blood proteins such as albumin [119]. Albumin also uses the FcRn recycling mechanisms of IgG so providing that an albumin specific sdAb does not interfere with this interaction it has the potential to improve pharmacokinetics of influenza specific sdAbs. This approach has as yet not been described for prophylactic or therapeutic applications of influenza specific sdAb but may have an advantage in mitigating possible or perceived risk of Fc related antibody dependent enhancement (ADE) of influenza [123,124,125].

## 5. Delivery of Single Domain Antibodies against Influenza for Therapy and Prophylaxis

Although vaccination is the main countermeasure against both seasonal and pandemic influenza their slow production and low effectiveness in certain vulnerable groups present challenges. Therefore, alternative approaches based on passive immunotherapy with monoclonal antibodies are of considerable interest with several currently in clinical development [125].

The delivery options of sdAbs for passive immunotherapy of influenza are intimately linked to their format, stability, pharmacokinetics and efficacy. Intravenous (IV), subcutaneous (SC), intranasal (IN) or oral delivery are all options that can be considered with appropriately designed sdAbs. Intranasal delivery is an attractive option particularly for respiratory diseases like influenza as the drug is delivered directly to the site of infection and can provide immediate effect. Intranasal delivery of any biological presents considerable challenges in maintaining sufficient activity in the harsh environment of the respiratory tract [126]. Single domain antibodies have well documented stability in extremes of temperature and pH so are well suited to formulation for inhaled delivery [94,126]. Intranasal delivery of a bivalent sdAb H5-VHHm against H5N1 has been shown to be capable of protecting mice from a lethal challenge with influenza virus both prophylactically and therapeutically [83].

Due to their small size [118,119] sdAbs require considerable improvement to their pharmacokinetics in vivo for either IV or SC delivery into the systemic circulation. The efficacy of Fc fusions of anti-influenza sdAbs against influenza HA and NA delivered intravenously has been demonstrated in animal challenge models in both therapeutic and prophylactic settings [74,89,109]. For longer term prophylaxis repeat injections would be required which might not be cost-effective and would limit widespread use. To overcome this limitation and provide more sustained long-term delivery of broadly neutralizing antibodies in vivo, antibody gene therapy is of considerable interest [99]. Gene therapy mediated antibody delivery using DNA, RNA or viral vectors can, in principle, provide ‘life-long’ expression of antibodies in the patient. This approach could benefit vulnerable patient groups such as the immune compromised or elderly who do not respond well to current influenza vaccines [62]. This approach is clearly very different to conventional vaccination because the immunity generated is independent of the host’s natural or vaccine induced immune response to influenza virus. The earliest studies have been done using conventional monoclonal antibodies and it was demonstrated that adeno-associated viral (AAV) vectors could be used to deliver sustained expression of broadly neutralizing antibodies against the HA stem region without prior exposure to influenza [108]. Both intramuscular expression [127] and intranasal expression [128] of broadly neutralizing HA stem binding conventional mAbs have been shown to be protective in mouse challenge models of influenza. Intranasal delivery has the advantage that antibody expression is in the respiratory tract directly at the site of infection but has the disadvantage that expression levels beyond the lungs are low and decrease over time due to turnover of nasal epithelial cells. Intramuscular delivery on the other hand gives much higher levels of expression in the systemic circulation which is more durable as muscle cells are quiescent and do not get replaced [127]. These early studies were conducted with conventional mAbs comprising separate light chain and heavy chain genes. Due to the limitations on size and complexity of genetic information that can be packaged into gene therapy vectors smaller simpler transgenes based on a single domain format and single open reading frame offer considerable advantages. The earliest study describing a sdAb format used adenovirus rather than AAV to deliver a subtype-specific sdAb against H5N1 converted into a bivalent format using a leucine zipper motif [129]. Following intranasal (IN) delivery this was shown to be protective in a mouse challenge model of influenza [129]. More recent studies have taken full advantage of the sdAb format and its compatibility with AAV mediated gene delivery. In a landmark study in 2018, Laursen and colleagues showed that intranasal AAV delivery of a multi-domain sdAb Fc fusion gave almost complete protection from all influenza subtypes in mouse challenge models [74]. In a more recent study, a similar broadly neutralizing R1a-B6-Fc fusion protein was evaluated using intramuscular AAV delivery with the aim to drive higher more sustainable levels of expression. This study similarly was able to show complete protection from lethal challenge of both H5N1 and H1N1 virus in mice [109]. The authors also showed that Fc function and ADCC was not essential for efficacy which had previously been proposed as essential for efficacy in this delivery format. This contrasts with previous reports of HA stem-binding antibodies requiring ADCC for maximum efficacy [115,116]. The authors speculated that this was due to the very high levels of expression possible with IM AAV gene delivery so compensating for any reduction in potency that may be seen in the absence of ADCC function [74,128]. In addition, a monovalent version of R1a-B6 was also shown to delay the onset of infection by 3 days which suggested that the high-level continuous production in muscle cells was also able to partially offset the rapid clearance [109]. Others have used mRNA instead of viral vectors to deliver a bispecific M2e sdAb locally to the respiratory tract in lipid particles and showed efficacy in a mouse challenge model [96].

## 6. Applications of sdAbs to Influenza Analytical Testing

There are surprisingly few reports using single domain antibodies for detecting viral antigens, including the detection of influenza virus. While nucleic acid based diagnostic methods provide high sensitivity, conventional antibodies or sdAb could be applied in antigen tests and in point-of-care devices that would offer unparalleled speed of detection [130]. The variability of the main viral antigens means that robust and durable assays would require broadly reactive antibodies or face the challenge of having to be updated frequently. A few ELISA-type assays for the detection of influenza viruses of various subtypes using sdAbs have been described [131,132,133]. The principles of these tests are similar, but the sdAbs used and the formats vary. They generally employ two non-competing sdAbs in a sandwich format, with the capture reagent tethered to a solid phase, such as an ELISA plate or magnetic beads, through biotin-streptavidin interaction, and a detection antibody linked to an enzyme enabling signal generation. However, these assays are subtype- or strain-specific and evidence of broad applicability beyond the strain or subtype chosen for the initial selection of the sdAbs used has not been reported. In a different approach, an engineered sdAb with a mouse IgE-Fc was used in a cell-based sensor; again, no data on breadth of reactivity and applicability was provided [134]. Another cell sensor-type assay used two sdAbs against the viral NP; one sdAb was expressed in the cell as a fusion protein with a DNA-binding domain, the other was fused to a transcription activation domain; upon infection of the cell with influenza virus, NP acted as a scaffold to assemble a bipartite transcription factor, leading to the expression of a reporter gene [135]. Due to the conserved nature of the NP, this system was able to detect viruses of three subtypes (H3, H5, H7). sdAbs have also been described as tools in serology testing and a sdAb targeting the viral NP protein was used in an antibody-blocking ELISA to detect antibodies against swine influenza viruses in serum samples [136]. While this assay has the theoretical potential to detect antibodies, suggesting prior infection, against any of the swine influenza subtypes (H1N1, H1N2, H3N2), a specificity assessment was not described.

**Figure 4:**
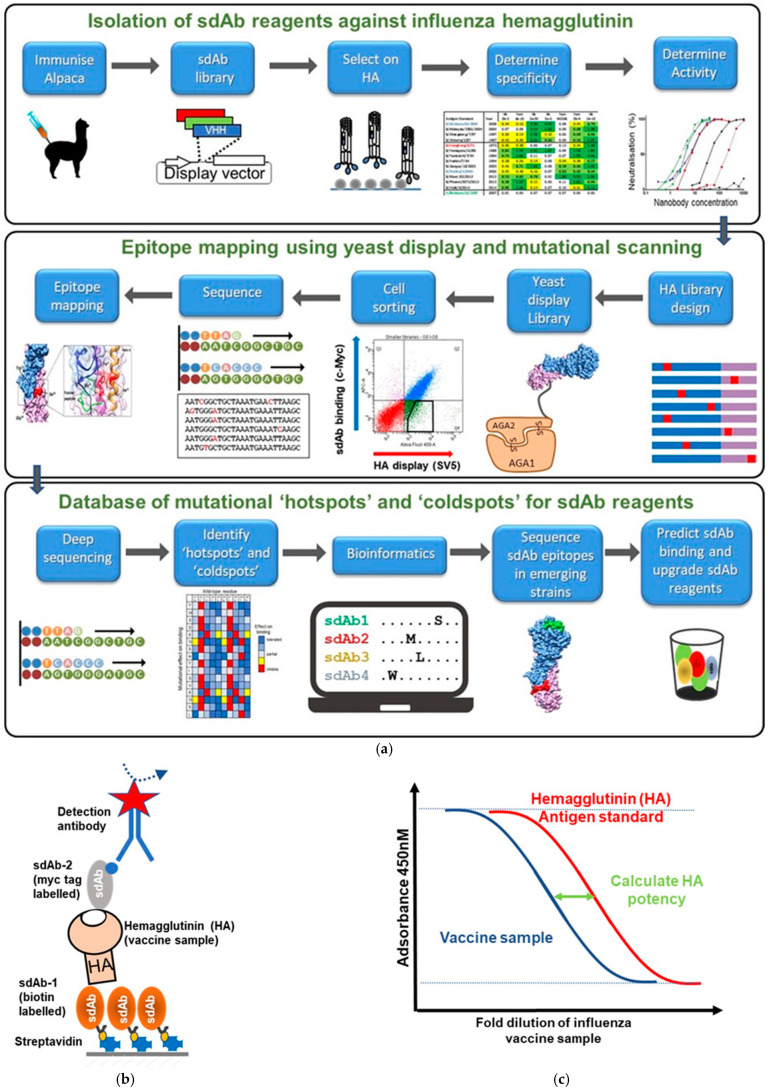
Isolation and epitope mapping of single domain antibodies against influenza hemagglutinin (HA) and applications in vaccine potency testing. (**a**) Adapted from [73]. Generation of panels of high affinity sdAbs against influenza HA using immunisation of alpacas with HA from key group 1, group 2 IAV and IBV strains. Construction of a sdAb (nanobody) display library and selection for antigen specific sdAbs with both broad reactivity and lineage specific binding for use in assessment of influenza vaccine potency assays are followed by epitope mapping using yeast display and mutational scanning, which includes design of a library of HA variants, display of the library on the yeast cell surface, selection using flow cytometric cell sorting to enrich HA variants that lose binding to sdAbs but retain display of correctly folded HA on yeast cell surface. Functional loss of binding is experimentally determined to confirm residues that are energetically important and contribute to the sdAb epitope. The epitope is then correlated with HA structure. Further analysis through deep sequencing of selection outputs can then be used to identify mutational ‘hotspots’ and also ‘coldspots’ where mutations are predicted to have little effect on binding of sdAb reagents. The frequencies of mutations at each position in HA relative to the non-selected population can be determined using bioinformatic analysis. It is envisaged that this approach can be used to generate a database of epitopes corresponding to a diverse collection of sdAbs recognising HA, which upon the emergence of a new viral strain can be used to predict which sdAbs could be chosen as suitable binding reagents for applications such as diagnosis, research, immune surveillance and vaccine potency testing. (**b**) ELISA assay for vaccine potency testing: A sandwich pair of sdAbs is used to measure HA content in influenza vaccines samples. sdAb-1 is biotin labelled and after coating onto a streptavidin ELISA plate is used as a conformational specific capture reagent for HA either from a vaccine sample or an influenza antigen standard with a known content of antigenically active HA. sdAb-2 is the detecting reagent binding to the HA captured on the ELISA plate. sdAb is labelled with a myc epitope tag which is used to detect binding. (**c**) A schematic representation of a vaccine potency assay readout and how it is used to evaluate HA content using comparison to a standard curve containing a known quantity of HA.

One emerging application for sdAbs is in the area of vaccine potency testing which is a crucial test used by vaccine manufacturers in the formulation and timely release of seasonal vaccines, as well as by independent control laboratories during batch release of vaccines before their release onto the market. This is a tightly controlled process underpinned by regulatory guidelines, which ensures that every year seasonal vaccines are available to protect public health. Since the 1970s, the single radial immunodiffusion (SRID) assay has been used to standardize and quantitate HA content in vaccines [137]. However, due to the dependence on strain-specific polyclonal sheep sera, which can take 2-3 months to generate and can be in limited supply, there has been an increasing call for alternative assays which are more sensitive and can be implemented more quickly. Broadly reactive antibodies which maintain reactivity over many seasons are of considerable interest, and sdAbs, because of their robust and simple structure, are ideal analytical reagents for this purpose. Single domain antibodies to diverse epitopes on different subtypes have been formatted for a sandwich and a competition ELISA and have been investigated for their potential in reliably quantifying HA content in vaccines (manuscript in preparation). Other assay formats based on protein arrays [138], surface plasmon resonance [139,140] or immunocapture [141,142] may be equally compatible with using cross-reactive sdAbs. To mitigate the risk of an individual sdAb losing reactivity with newly emerging influenza virus antigenic variants, cocktails of sdAbs recognizing non-overlapping epitopes could be used in potency testing, as it is highly unlikely that changes in the HA prompting an update of the influenza vaccine would affect several sdAb epitopes at once. Several sdAbs to the HA of different influenza subtypes with both broad reactivity and lineage specific reactivity have been isolated and their epitopes correlated with their specificity using yeast display mutational scanning [73]. This technique allows the rapid high-throughput testing in parallel libraries of point mutations for their effect on binding of influenza specific sdAb reagents [73,75,81]. In addition, it is possible to identify mutational ‘coldspots’ which are residues which if mutated are predicted to have little effect on sdAb binding [73]. This profiling and experimental testing of the effect of multiple mutations on sdAb binding may be used to identify, as soon as a new strain emerges, which sdAbs may be impacted and which are not and also aid the generation of sdAb cocktails for seasonal vaccine release [73,75,81] (Figure 4). If such analysis suggests the need to update the composition of sdAb cocktails or of individual sdAbs used in potency testing, a pipeline to quickly identify new sdAb can be established, based on libraries of sdAbs and a combination of efficient selection protocols and next generation sequencing approaches (Figure 4).

## 7. Conclusions and Perspectives

The rediscovery of HA stem-binding, broadly neutralizing antibodies in 2008 catalyzed the isolation of many different human monoclonal antibodies and the characterization of several highly conserved epitopes on the influenza virus. The limitations of conventional monoclonal antibodies have driven considerable interest in single domain antibodies owing to their small size, flexible formatting and high stability. In addition, the isolation of sdAbs from camelids using display technologies means there is a ready and unlimited supply of high affinity recombinant reagents. These advantages lend themselves to the unique challenges of generating therapeutic antibodies to a rapidly evolving pathogen like influenza virus. Single domain antibodies with broad reactivity against influenza have been discovered which bind to all three influenza viral surface proteins as well as nucleoprotein. The mechanisms of action, epitope and format can be readily adapted for different applications to enhance potency, delivery, cross-reactivity, production, stability, effector function, pharmacokinetics and immunogenicity. Furthermore, the same properties of sdAbs mean that they suit applications in diagnostics, vaccine potency assays and influenza research. These reagents could be produced rapidly when an antigenically novel influenza strain emerges.

Although much is known about neutralizing sdAbs against influenza several questions remain to be answered. For example: how do the binding and neutralization mechanisms of sdAbs compare to conventional antibodies targeting similar conserved regions of the virus? Do differences translate into therapeutic advantages, for example through increased resistance to mutational escape? In addition to targeting conserved epitopes, the ability to fuse several sdAbs together specific for different epitopes is expected to reduce the emergence of resistant viruses. Multi-specific sdAbs have a clear advantage over bispecific formats based on conventional IgGs which require the complex expression and assembly of up to four polypeptides. Much simpler sdAb single gene constructs may have a considerable advantage for gene therapy mediated antibody delivery where there are limits on the size and complexity of genes which can be packaged into viral vectors. With further understanding of the risks of mutational escape, immunogenicity and antibody dependent enhancement of influenza (ADE) it is expected that optimal therapeutics based on the sdAb format can be designed and thus translate their early promise into cost-effective delivery of passive immunotherapy to vulnerable patient groups.

## References

[1] Santos-Preciado J., Franco-Paredes C., Hernandez-Flores I., Tellez I., Del Rio C., Tapia-Conyer R. (2009). What have we learned from the novel influenza A (H1N1) pandemic in 2009 for strengthening pandemic influenza preparedness?. Arch. Med. Res..

[2] Girard M.P., Tam J.S., Assossou O.M., Kieny M.P. (2010). The 2009 A (H1N1) influenza virus pandemic: A review. Vaccine.

[3] Carrat F., Flahault A. (2007). Influenza vaccine: The challenge of antigenic drift. Vaccine.

[4] Yamayoshi S., Kawaoka Y. (2019). Current and future influenza vaccines. Nat. Med..

[5] Webster R.G., Laver W.G., Air G.M., Schild G.C. (1982). Molecular mechanisms of variation in influenza viruses. Nature.

[6] Air G.M. (2015). Influenza virus antigenicity and broadly neutralizing epitopes. Curr. Opin. Virol..

[7] Marasco W.A., Sui J. (2007). The growth and potential of human antiviral monoclonal antibody therapeutics. Nat. Biotechnol..

[8] Nabel G.J., Fauci A.S. (2010). Induction of unnatural immunity: Prospects for a broadly protective universal influenza vaccine. Nat. Med..

[9] Wei C.J., Crank M.C., Shiver J., Graham B.S., Mascola J.R., Nabel G.J. (2020). Author Correction: Next-generation influenza vaccines: Opportunities and challenges. Nat. Rev. Drug Discov..

[10] Wei C.J., Crank M.C., Shiver J., Graham B.S., Mascola J.R., Nabel G.J. (2020). Next-generation influenza vaccines: Opportunities and challenges. Nat. Rev. Drug Discov..

[11] Epstein S.L., Lo C.Y., Misplon J.A., Lawson C.M., Hendrickson B.A., Max E.E., Subbarao K. (1997). Mechanisms of heterosubtypic immunity to lethal influenza A virus infection in fully immunocompetent, T cell-depleted, beta2-microglobulin-deficient, and J chain-deficient mice. J. Immunol..

[12] Epstein S.L., Price G.E. (2010). Cross-protective immunity to influenza A viruses. Expert Rev. Vaccines.

[13] Okuno Y., Isegawa Y.U., Sasao F., Ueda S. (1993). A common neutralizing epitope conserved between the hemagglutinins of influenza A virus H1 and H2 strains. J. Virol..

[14] Ohmit S.E., Petrie J.G., Cross R.T., Johnson E., Monto A.S. (2011). Influenza hemagglutination-inhibition antibody titer as a correlate of vaccine-induced protection. J. Infect. Dis..

[15] Dreyfus C., Ekiert D.C., Wilson I.A. (2013). Structure of a classical broadly neutralizing stem antibody in complex with a pandemic H2 influenza virus hemagglutinin. J. Virol..

[16] Throsby M., van den Brink E., Jongeneelen M., Poon L.L., Alard P., Cornelissen L., Bakker A., Cox F., van Deventer E., Guan Y. (2008). Heterosubtypic neutralizing monoclonal antibodies cross-protective against H5N1 and H1N1 recovered from human IgM+ memory B cells. PLoS ONE.

[17] Ekiert D.C., Bhabha G., Elsliger M.A., Friesen R.H., Jongeneelen M., Throsby M., Goudsmit J., Wilson I.A. (2009). Antibody recognition of a highly conserved influenza virus epitope. Science.

[18] Sui J., Hwang W.C., Perez S., Wei G., Aird D., Chen L.M., Santelli E., Stec B., Cadwell G., Ali M. (2009). Structural and functional bases for broad-spectrum neutralization of avian and human influenza A viruses. Nat. Struct. Mol. Biol..

[19] Ekiert D.C., Friesen R.H., Bhabha G., Kwaks T., Jongeneelen M., Yu W., Ophorst C., Cox F., Korse H.J., Brandenburg B. (2011). A highly conserved neutralizing epitope on group 2 influenza A viruses. Science.

[20] Corti D., Lanzavecchia A. (2013). Broadly neutralizing antiviral antibodies. Annu. Rev. Immunol..

[21] Traggiai E., Becker S., Subbarao K., Kolesnikova L., Uematsu Y., Gismondo M.R., Murphy B.R., Rappuoli R., Lanzavecchia A. (2004). An efficient method to make human monoclonal antibodies from memory B cells: Potent neutralization of SARS coronavirus. Nat. Med..

[22] Hoogenboom H.R., de Bruıne A.P., Hufton S.E., Hoet R.M., Arends J.W., Roovers R.C. (1998). Antibody phage display technology and its applications. Immunotechnology.

[23] Kashyap A.K., Steel J., Oner A.F., Dillon M.A., Swale R.E., Wall K.M., Perry K.J., Faynboym A., Ilhan M., Horowitz M. (2008). Combinatorial antibody libraries from survivors of the Turkish H5N1 avian influenza outbreak reveal virus neutralization strategies. Proc. Natl. Acad. Sci. USA.

[24] Ekiert D.C., Kashyap A.K., Steel J., Rubrum A., Bhabha G., Khayat R., Lee J.H., Dillon M.A., O’Neil R.E., Faynboym A.M. (2012). Cross-neutralization of influenza A viruses mediated by a single antibody loop. Nature.

[25] Lang S., Xie J., Zhu X., Wu N.C., Lerner R.A., Wilson I.A. (2017). Antibody 27F3 Broadly Targets Influenza A Group 1 and 2 Hemagglutinins through a Further Variation in VH1-69 Antibody Orientation on the HA Stem. Cell Rep..

[26] Lee P.S., Ohshima N., Stanfield R.L., Yu W., Iba Y., Okuno Y., Kurosawa Y., Wilson I.A. (2014). Receptor mimicry by antibody F045-092 facilitates universal binding to the H3 subtype of influenza virus. Nat. Commun..

[27] Kwong P.D., Wilson I.A. (2009). HIV-1 and influenza antibodies: Seeing antigens in new ways. Nat. Immunol..

[28] Zhou T., Xu L., Dey B., Hessell A.J., Van Ryk D., Xiang S.H., Yang X., Zhang M.Y., Zwick M.B., Arthos J. (2007). Structural definition of a conserved neutralization epitope on HIV-1 gp120. Nature.

[29] Arbabi Ghahroudi M., Desmyter A., Wyns L., Hamers R., Muyldermans S. (1997). Selection and identification of single domain antibody fragments from camel heavy-chain antibodies. FEBS Lett..

[30] Hamers-Casterman C.T., Atarhouch T., Muyldermans S., Robinson G., Hammers C., Songa E.B., Bendahman N., Hammers R. (1993). Naturally occurring antibodies devoid of light chains. Nature.

[31] Streltsov V.A., Carmichael J.A., Nuttall S.D. (2005). Structure of a shark IgNAR antibody variable domain and modeling of an early-developmental isotype. Protein Sci..

[32] Streltsov V.A., Varghese J.N., Carmichael J.A., Irving R.A., Hudson P.J., Nuttall S.D. (2004). Structural evidence for evolution of shark Ig new antigen receptor variable domain antibodies from a cell-surface receptor. Proc. Natl. Acad. Sci. USA..

[33] Muyldermans S., Baral T.N., Retamozzo V.C., De Baetselier P., De Genst E., Kinne J., Leonhardt H., Magez S., Nguyen V.K., Revets H. (2009). Camelid immunoglobulins and nanobody technology. Vet. Immunol. Immunopathol..

[34] Vu K.B., Ghahroudi M.A., Wyns L., Muyldermans S. (1997). Comparison of llama VH sequences from conventional and heavy chain antibodies. Mol. Immunol..

[35] Harmsen M.M., Ruuls R.C., Nijman I.J., Niewold T.A., Frenken L.G., de Geus B. (2000). Llama heavy-chain V regions consist of at least four distinct subfamilies revealing novel sequence features. Mol. Immunol..

[36] Henry K.A., MacKenzie C.R. (2018). Antigen recognition by single-domain antibodies: Structural latitudes and constraints. Mabs.

[37] Yan J., Li G., Hu Y., Ou W., Wan Y. (2014). Construction of a synthetic phage-displayed Nanobody library with CDR3 regions randomized by trinucleotide cassettes for diagnostic applications. J. Transl. Med..

[38] Moutel S., Bery N., Bernard V., Keller L., Lemesre E., de Marco A., Ligat L., Rain J.C., Favre G., Olichon A. (2016). NaLi-H1: A universal synthetic library of humanized nanobodies providing highly functional antibodies and intrabodies. Elife.

[39] Wei G., Meng W., Guo H., Pan W., Liu J., Peng T., Chen L., Chen C.Y. (2011). Potent neutralization of influenza A virus by a single-domain antibody blocking M2 ion channel protein. PLoS ONE.

[40] Ward E.S., Güssow D., Griffiths A.D., Jones P.T., Winter G. (1989). Binding activities of a repertoire of single immunoglobulin variable domains secreted from Escherichia coli. Nature.

[41] Chen W., Zhu Z., Feng Y., Dimitrov D.S. (2008). Human domain antibodies to conserved sterically restricted regions on gp120 as exceptionally potent cross-reactive HIV-1 neutralizers. Proc. Natl. Acad. Sci. USA.

[42] Van Den Beucken T., van Neer N., Sablon E., Desmet J., Celis L., Hoogenboom H.R., Hufton S.E. (2001). Building novel binding ligands to B7.1 and B7.2 based on human antibody single variable light chain domains. J. Mol. Biol..

[43] Vanlandschoot P., Stortelers C., Beirnaert E., Ibañez L.I., Schepens B., Depla E., Saelens X. (2011). Nanobodies(R): New ammunition to battle viruses. Antiviral Res..

[44] De Genst E., Silence K., Decanniere K., Conrath K., Loris R., Kinne J., Muyldermans S., Wyns L. (2006). Molecular basis for the preferential cleft recognition by dromedary heavy-chain antibodies. Proc. Natl. Acad. Sci. USA.

[45] Mitchell L.S., Colwell L.J. (2018). Comparative analysis of nanobody sequence and structure data. Proteins.

[46] Strauss M., Schotte L., Thys B., Filman D.J., Hogle J.M. (2016). Five of Five VHHs Neutralizing Poliovirus Bind the Receptor-Binding Site. J. Virol..

[47] Garza J.A., Taylor A.B., Sherwood L.J., Hart P.J., Hayhurst A. (2017). Unveiling a Drift Resistant Cryptotope within Marburgvirus Nucleoprotein Recognized by Llama Single-Domain Antibodies. Front. Immunol..

[48] Zavrtanik U., Lukan J., Loris R., Lah J., Hadži S. (2018). Structural Basis of Epitope Recognition by Heavy-Chain Camelid Antibodies. J. Mol. Biol..

[49] Rossey I., Gilman M.S., Kabeche S.C., Sedeyn K., Wrapp D., Kanekiyo M., Chen M., Mas V., Spitaels J., Melero J.A. (2017). Potent single-domain antibodies that arrest respiratory syncytial virus fusion protein in its prefusion state. Nat. Commun..

[50] Klein J.S., Gnanapragasam P.N., Galimidi R.P., Foglesong C.P., West A.P., Bjorkman P.J. (2009). Examination of the contributions of size and avidity to the neutralization mechanisms of the anti-HIV antibodies b12 and 4E10. Proc. Natl. Acad. Sci. USA.

[51] Kringelum J.V., Nielsen M., Padkjær S.B., Lund O. (2013). Structural analysis of B-cell epitopes in antibody: Protein complexes. Mol. Immunol..

[52] Rubinstein N.D., Mayrose I., Halperin D., Yekutieli D., Gershoni J.M., Pupko T. (2008). Computational characterization of B-cell epitopes. Mol. Immunol..

[53] Thornburg N.J., Zhang H., Bangaru S., Sapparapu G., Kose N., Lampley R.M., Bombardi R.G., Yu Y., Graham S., Branchizio A. (2016). H7N9 influenza virus neutralizing antibodies that possess few somatic mutations. J. Clin. Investig..

[54] Huang K.Y., Rijal P., Jiang H., Wang B., Schimanski L., Dong T., Liu Y.M., Chang P., Iqbal M., Wang M.C. (2019). Structure-function analysis of neutralizing antibodies to H7N9 influenza from naturally infected humans. Nat. Microbiol..

[55] Avnir Y., Tallarico A.S., Zhu Q., Bennett A.S., Connelly G., Sheehan J., Sui J., Fahmy A., Huang C.Y., Cadwell G. (2014). Molecular signatures of hemagglutinin stem-directed heterosubtypic human neutralizing antibodies against influenza A viruses. PLoS Pathog..

[56] Wang R., Song A., Levin J., Dennis D., Zhang N.J., Yoshida H., Koriazova L., Madura L., Shapiro L., Matsumoto A. (2008). Therapeutic potential of a fully human monoclonal antibody against influenza A virus M2 protein. Antiviral Res..

[57] Beerli R.R., Bauer M., Schmitz N., Buser R.B., Gwerder M., Muntwiler S., Renner W.A., Saudan P., Bachmann M.F. (2009). Prophylactic and therapeutic activity of fully human monoclonal antibodies directed against influenza A M2 protein. Virol. J..

[58] Doyle T.M., Hashem A.M., Li C., Van Domselaar G., Larocque L., Wang J., Smith D., Cyr T., Farnsworth A., He R. (2013). Universal anti-neuraminidase antibody inhibiting all influenza A subtypes. Antiviral Res..

[59] Gravel C., Li C., Wang J., Hashem A.M., Jaentschke B., Xu K.W., Lorbetskie B., Gingras G., Aubin Y., Van Domselaar G. (2010). Qualitative and quantitative analyses of virtually all subtypes of influenza A and B viral neuraminidases using antibodies targeting the universally conserved sequences. Vaccine.

[60] LaMere M.W., Lam H.T., Moquin A., Haynes L., Lund F.E., Randall T.D., Kaminski D.A. (2011). Contributions of antinucleoprotein IgG to heterosubtypic immunity against influenza virus. J. Immunol..

[61] Skehel J.J., Wiley D.C. (2000). Receptor binding and membrane fusion in virus entry: The influenza hemagglutinin. Annu. Rev. Biochem..

[62] Russell R.J., Gamblin S.J., Haire L.F., Stevens D.J., Xiao B., Ha Y., Skehel J.J. (2004). H1 and H7 influenza haemagglutinin structures extend a structural classification of haemagglutinin subtypes. Virology.

[63] Giotis E.S., Carnell G., Young E.F., Ghanny S., Soteropoulos P., Wang L.F., Barclay W.S., Skinner M.A., Temperton N. (2019). Entry of the bat influenza H17N10 virus into mammalian cells is enabled by the MHC class II HLA-DR receptor. Nat. Microbiol..

[64] Paul Glezen W., Schmier J.K., Kuehn C.M., Ryan K.J., Oxford J. (2013). The burden of influenza B: A structured literature review. Am. J. Public Health.

[65] Rota P.A., Wallis T.R., Harmon M.W., Rota J.S., Kendal A.P., Nerome K. (1990). Cocirculation of two distinct evolutionary lineages of influenza type B virus since 1983. Virology.

[66] Weir J.P., Gruber M.F. (2016). An overview of the regulation of influenza vaccines in the United States. Influenza Other Respir. Viruses.

[67] Wu N.C., Wilson I.A. (2017). A Perspective on the Structural and Functional Constraints for Immune Evasion: Insights from Influenza Virus. J. Mol. Biol..

[68] Benton D.J., Gamblin S.J., Rosenthal P.B., Skehel J.J. (2020). Structural transitions in influenza haemagglutinin at membrane fusion pH. Nature.

[69] Corti D., Voss J., Gamblin S.J., Codoni G., Macagno A., Jarrossay D., Vachieri S.G., Pinna D., Minola A., Vanzetta F. (2011). A neutralizing antibody selected from plasma cells that binds to group 1 and group 2 influenza A hemagglutinins. Science.

[70] Dreyfus C., Laursen N.S., Kwaks T., Zuijdgeest D., Khayat R., Ekiert D.C., Lee J.H., Metlagel Z., Bujny M.V., Jongeneelen M. (2012). Highly conserved protective epitopes on influenza B viruses. Science.

[71] Russell R.J., Kerry P.S., Stevens D.J., Steinhauer D.A., Martin S.R., Gamblin S.J., Skehel J.J. (2008). Structure of influenza hemagglutinin in complex with an inhibitor of membrane fusion. Proc. Natl. Acad. Sci. USA.

[72] Hufton S.E., Risley P., Ball C.R., Major D., Engelhardt O.G., Poole S. (2014). The breadth of cross sub-type neutralisation activity of a single domain antibody to influenza hemagglutinin can be increased by antibody valency. PLoS ONE.

[73] Gaiotto T., Hufton S.E. (2016). Cross-Neutralising Nanobodies Bind to a Conserved Pocket in the Hemagglutinin Stem Region Identified Using Yeast Display and Deep Mutational Scanning. PLoS ONE.

[74] Laursen N.S., Friesen R.H., Zhu X., Jongeneelen M., Blokland S., Vermond J., van Eijgen A., Tang C., van Diepen H., Obmolova G. (2018). Universal protection against influenza infection by a multidomain antibody to influenza hemagglutinin. Science.

[75] Gaiotto T., Ramage W., Ball C., Risley P., Carnell G.W., Temperton N., Engelhardt O.G., Hufton S.E. (2021). Nanobodies mapped to cross-reactive and divergent epitopes on A(H7N9) influenza hemagglutinin using yeast display. Sci. Rep..

[76] Zhang Y., Xu C., Zhang H., Liu G.D., Xue C., Cao Y. (2019). Targeting Hemagglutinin: Approaches for Broad Protection against the Influenza A Virus. Viruses.

[77] Petrova N.V., Russell C.A. (2018). The evolution of seasonal influenza viruses. Nat. Rev. Microbiol..

[78] Koel B.F., Burke D.F., Bestebroer T.M., Van Der Vliet S., Zondag G.C., Vervaet G., Skepner E., Lewis N.S., Spronken M.I., Russell C.A. (2013). Substitutions near the receptor binding site determine major antigenic change during influenza virus evolution. Science.

[79] Linster M., Schrauwen E.J., van der Vliet S., Burke D.F., Lexmond P., Bestebroer T.M., Smith D.J., Herfst S., Koel B.F., Fouchier R.A. (2019). The Molecular Basis for Antigenic Drift of Human A/H2N2 Influenza Viruses. J. Virol..

[80] Krammer F., Palese P. (2019). Universal Influenza Virus Vaccines That Target the Conserved Hemagglutinin Stalk and Conserved Sites in the Head Domain. J. Infect. Dis..

[81] Ramage W., Gaiotto T., Ball C., Risley P., Carnell G.W., Temperton N., Cheung C.Y., Engelhardt O.G., Hufton S.E. (2019). Cross-Reactive and Lineage-Specific Single Domain Antibodies against Influenza B Hemagglutinin. Antibodies.

[82] Tillib S.V., Ivanova T.I., Vasilev L.A., Rutovskaya M.V., Saakyan S.A., Gribova I.Y., Tutykhina I.L., Sedova E.S., Lysenko A.A., Shmarov M.M. (2013). Formatted single-domain antibodies can protect mice against infection with influenza virus (H5N2). Antiviral Res..

[83] Ibanez L.I., De Filette M., Hultberg A., Verrips T., Temperton N., Weiss R.A., Vandevelde W., Schepens B., Vanlandschoot P., Saelens X. (2011). Nanobodies with in vitro neutralizing activity protect mice against H5N1 influenza virus infection. J. Infect. Dis..

[84] Xu R., Ekiert D.C., Krause J.C., Hai R., Crowe J.E., Wilson I.A. (2010). Structural basis of preexisting immunity to the 2009 H1N1 pandemic influenza virus. Science.

[85] Zhang Y., Aevermann B.D., Anderson T.K., Burke D.F., Dauphin G., Gu Z., He S., Kumar S., Larsen C.N., Lee A.J. (2017). Influenza Research Database: An integrated bioinformatics resource for influenza virus research. Nucleic Acids Res..

[86] Friesen R.H., Lee P.S., Stoop E.J., Hoffman R.M., Ekiert D.C., Bhabha G., Yu W., Juraszek J., Koudstaal W., Jongeneelen M. (2014). A common solution to group 2 influenza virus neutralization. Proc. Natl. Acad. Sci. USA.

[87] Krissinel E., Henrick K. (2007). Inference of macromolecular assemblies from crystalline state. J. Mol. Biol..

[88] Wohlbold T.J., Podolsky K.A., Chromikova V., Kirkpatrick E., Falconieri V., Meade P., Amanat F., Tan J., Tan G.S., Subramaniam S. (2017). Broadly protective murine monoclonal antibodies against influenza B virus target highly conserved neuraminidase epitopes. Nat. Microbiol..

[89] Cardoso F.M., Ibañez L.I., Van den Hoecke S., De Baets S., Smet A., Roose K., Schepens B., Descamps F.J., Fiers W., Muyldermans S. (2014). Single-domain antibodies targeting neuraminidase protect against an H5N1 influenza virus challenge. J. Virol..

[90] Harmsen M.M., Blokker J.C., Pritz-Verschuren S.B., Bartelink W., Van der Burg H., Koch G. (2013). Isolation of Panels of Llama Single-Domain Antibody Fragments Binding All Nine Neuraminidase subtypes of Influenza A Virus. Antibodies.

[91] Pielak R.M., Chou J.J. (2011). Influenza M2 proton channels. Biochim. Biophys. Acta.

[92] Yamauchi Y. (2020). Influenza A virus uncoating. Adv. Virus Res..

[93] Wang Y., Zhou L., Shi H., Xu H., Yao H., Xi X.G., Toyoda T., Wang X., Wang T. (2009). Monoclonal antibody recognizing SLLTEVET epitope of M2 protein potently inhibited the replication of influenza A viruses in MDCK cells. Biochem. Biophys. Res. Commun..

[94] De Vlieger D., Hoffmann K., Van Molle I., Nerinckx W., Van Hoecke L., Ballegeer M., Creytens S., Remaut H., Hengel H., Schepens B. (2019). Selective Engagement of FcgammaRIV by a M2e-Specific Single Domain Antibody Construct Protects against Influenza a Virus Infection. Front. Immunol..

[95] Van Hoecke L., Verbeke R., De Vlieger D., Dewitte H., Roose K., Van Nevel S., Krysko O., Bachert C., Schepens B., Lentacker I. (2020). mRNA Encoding a Bispecific Single Domain Antibody Construct Protects against Influenza A Virus Infection in Mice. Mol. Ther. Nucleic Acids.

[96] Hanke L., Knockenhauer K.E., Brewer R.C., van Diest E., Schmidt F.I., Schwartz T.U., Ploegh H.L. (2016). The Antiviral Mechanism of an Influenza A Virus Nucleoprotein-Specific Single-Domain Antibody Fragment. Mbio.

[97] Ashour J., Schmidt F.I., Hanke L., Cragnolini J., Cavallari M., Altenburg A., Brewer R., Ingram J., Shoemaker C., Ploegh H.L. (2015). Intracellular expression of camelid single-domain antibodies specific for influenza virus nucleoprotein uncovers distinct features of its nuclear localization. J. Virol..

[98] Schmidt F.I., Hanke L., Morin B., Brewer R., Brusic V., Whelan S.P., Ploegh H.L. (2016). Phenotypic lentivirus screens to identify functional single domain antibodies. Nat. Microbiol..

[99] Deal C.E., Balazs A.B. (2015). Engineering humoral immunity as prophylaxis or therapy. Curr. Opin. Immunol..

[100] Lo A.Y., Zhu Q., Marasco W.A. (2008). Intracellular antibodies (intrabodies) and their therapeutic potential. Handb. Exp. Pharmacol..

[101] Verma S., Soto J., Vasudevan A., Schmeisser F., Alvarado-Facundo E., Wang W., Weiss C.D., Weir J.P. (2017). Determination of influenza B identity and potency in quadrivalent inactivated influenza vaccines using lineage-specific monoclonal antibodies. PLoS ONE.

[102] Sevy A.M., Gilchuk I.M., Brown B.P., Bozhanova N.G., Nargi R., Jensen M., Meiler J., Crowe J.E. (2020). Computationally Designed Cyclic Peptides Derived from an Antibody Loop Increase Breadth of Binding for Influenza Variants. Structure.

[103] Mitchell L.S., Colwell L.J. (2018). Analysis of nanobody paratopes reveals greater diversity than classical antibodies. Protein Eng. Des. Sel..

[104] Kallewaard N.L., Corti D., Collins P.J., Neu U., McAuliffe J.M., Benjamin E., Wachter-Rosati L., Palmer-Hill F.J., Yuan A.Q., Walker P.A. (2016). Structure and Function Analysis of an Antibody Recognizing All Influenza A Subtypes. Cell.

[105] Joyce M.G., Wheatley A.K., Thomas P.V., Chuang G.Y., Soto C., Bailer R.T., Druz A., Georgiev I.S., Gillespie R.A., Kanekiyo M. (2016). Vaccine-Induced Antibodies that Neutralize Group 1 and Group 2 Influenza A Viruses. Cell.

[106] Tan G.S., Lee P.S., Hoffman R.M., Mazel-Sanchez B., Krammer F., Leon P.E., Ward A.B., Wilson I.A., Palese P. (2014). Characterization of a broadly neutralizing monoclonal antibody that targets the fusion domain of group 2 influenza A virus hemagglutinin. J. Virol..

[107] Brandenburg B., Koudstaal W., Goudsmit J., Klaren V., Tang C., Bujny M.V., Korse H.J., Kwaks T., Otterstrom J.J., Juraszek J. (2013). Mechanisms of hemagglutinin targeted influenza virus neutralization. PLoS ONE.

[108] Hultberg A., Temperton N.J., Rosseels V., Koenders M., Gonzalez-Pajuelo M., Schepens B., Ibañez L.I., Vanlandschoot P., Schillemans J., Saunders M. (2011). Llama-Derived Single Domain Antibodies to Build Multivalent, Superpotent and Broadened Neutralizing Anti-Viral Molecules. PLoS ONE.

[109] Del Rosario J.M., Smith M., Zaki K., Risley P., Temperton N., Engelhardt O.G., Collins M., Takeuchi Y., Hufton S.E. (2020). Protection From Influenza by Intramuscular Gene Vector Delivery of a Broadly Neutralizing Nanobody Does Not Depend on Antibody Dependent Cellular Cytotoxicity. Front. Immunol..

[110] Fan K., Jiang B., Guan Z., He J., Yang D., Xie N., Nie G., Xie C., Yan X. (2018). Fenobody: A Ferritin-Displayed Nanobody with High Apparent Affinity and Half-Life Extension. Anal. Chem..

[111] Lee P.S., Yoshida R., Ekiert D.C., Sakai N., Suzuki Y., Takada A., Wilson I.A. (2012). Heterosubtypic antibody recognition of the influenza virus hemagglutinin receptor binding site enhanced by avidity. Proc. Natl. Acad. Sci. USA.

[112] Hayden F.G. (2006). Antiviral resistance in influenza viruses--implications for management and pandemic response. N. Engl. J. Med..

[113] Tharakaraman K., Subramanian V., Cain D., Sasisekharan V., Sasisekharan R. (2014). Broadly neutralizing influenza hemagglutinin stem-specific antibody CR8020 targets residues that are prone to escape due to host selection pressure. Cell Host Microbe.

[114] Howell K.A., Brannan J.M., Bryan C., McNeal A., Davidson E., Turner H.L., Vu H., Shulenin S., He S., Kuehne A. (2017). Cooperativity Enables Non-neutralizing Antibodies to Neutralize Ebolavirus. Cell Rep..

[115] DiLillo D.J., Palese P., Wilson P.C., Ravetch J.V. (2016). Broadly neutralizing anti-influenza antibodies require Fc receptor engagement for in vivo protection. J. Clin. Investig..

[116] DiLillo D.J., Tan G.S., Palese P., Ravetch J.V. (2014). Broadly neutralizing hemagglutinin stalk-specific antibodies require FcgammaR interactions for protection against influenza virus in vivo. Nat. Med..

[117] Vanderven H.A., Kent S.J. (2020). The protective potential of Fc-mediated antibody functions against influenza virus and other viral pathogens. Immunol. Cell Biol..

[118] Cortez-Retamozo V., Lauwereys M., Hassanzadeh Gh G., Gobert M., Conrath K., Muyldermans S., De Baetselier P., Revets H. (2002). Efficient tumor targeting by single-domain antibody fragments of camels. Int. J. Cancer.

[119] Hoefman S., Ottevaere I., Baumeister J., Sargentini-Maier M.L. (2015). Pre-Clinical Intravenous Serum Pharmacokinetics of Albumin Binding and Non-Half-Life Extended Nanobodies®. Antibodies.

[120] Batra S.K., Jain M., Wittel U.A., Chauhan S.C., Colcher D. (2002). Pharmacokinetics and biodistribution of genetically engineered antibodies. Curr. Opin. Biotechnol..

[121] Kontermann R.E. (2009). Strategies to extend plasma half-lives of recombinant antibodies. BioDrugs.

[122] Stapleton N.M., Andersen J.T., Stemerding A.M., Bjarnarson S.P., Verheul R.C., Gerritsen J., Zhao Y., Kleijer M., Sandlie I., De Haas M. (2011). Competition for FcRn-mediated transport gives rise to short half-life of human IgG3 and offers therapeutic potential. Nat. Commun..

[123] Khurana S., Loving C.L., Manischewitz J., King L.R., Gauger P.C., Henningson J., Vincent A.L., Golding H. (2013). Vaccine-induced anti-HA2 antibodies promote virus fusion and enhance influenza virus respiratory disease. Sci. Transl. Med..

[124] Ramakrishnan B., Viswanathan K., Tharakaraman K., Dančík V., Raman R., Babcock G.J., Shriver Z., Sasisekharan R. (2016). A Structural and Mathematical Modeling Analysis of the Likelihood of Antibody-Dependent Enhancement in Influenza. Trends Microbiol..

[125] Sparrow E., Friede M., Sheikh M., Torvaldsen S., Newall A.T. (2016). Passive immunization for influenza through antibody therapies, a review of the pipeline, challenges and potential applications. Vaccine.

[126] Van Heeke G., Allosery K., De Brabandere V., De Smedt T., Detalle L., de Fougerolles A. (2017). Nanobodies(R) as inhaled biotherapeutics for lung diseases. Pharmacol. Ther..

[127] Balazs A.B., Bloom J.D., Hong C.M., Rao D.S., Baltimore D. (2013). Broad protection against influenza infection by vectored immunoprophylaxis in mice. Nat. Biotechnol..

[128] Limberis M.P., Adam V.S., Wong G., Gren J., Kobasa D., Ross T.M., Kobinger G.P., Tretiakova A., Wilson J.M. (2013). Intranasal antibody gene transfer in mice and ferrets elicits broad protection against pandemic influenza. Sci. Transl. Med..

[129] Tutykhina I.L., Sedova E.S., Gribova I.Y., Ivanova T.I., Vasilev L.A., Rutovskaya M.V., Lysenko A.A., Shmarov M.M., Logunov D.Y., Naroditsky B.S. (2013). Passive immunization with a recombinant adenovirus expressing an HA (H5)-specific single-domain antibody protects mice from lethal influenza infection. Antiviral Res..

[130] Nelson P.P., Rath B.A., Fragkou P.C., Antalis E., Tsiodras S., Skevaki C. (2020). Current and Future Point-of-Care Tests for Emerging and New Respiratory Viruses and Future Perspectives. Front. Cell Infect. Microbiol..

[131] Gong X., Zhu M., Li G., Lu X., Wan Y. (2016). Specific determination of influenza H7N2 virus based on biotinylated single-domain antibody from a phage-displayed library. Anal. Biochem..

[132] Zhu M., Gong X., Hu Y., Ou W., Wan Y. (2014). Streptavidin-biotin-based directional double Nanobody sandwich ELISA for clinical rapid and sensitive detection of influenza H5N1. J. Transl. Med..

[133] Zhu M., Hu Y., Li G., Ou W., Mao P., Xin S., Wan Y. (2014). Combining magnetic nanoparticle with biotinylated nanobodies for rapid and sensitive detection of influenza H3N2. Nanoscale Res. Lett..

[134] Qu M., Boruah B.M., Zhang W., Li Y., Liu W., Bi Y., Gao G.F., Yang R., Liu D., Gao B. (2013). A Rat Basophilic Leukaemia cell sensor for the detection of pathogenic viruses. Biosens. Bioelectron..

[135] Cao J., Zhong N., Wang G., Wang M., Zhang B., Fu B., Wang Y., Zhang T., Zhang Y., Yang K. (2019). Nanobody-based sandwich reporter system for living cell sensing influenza A virus infection. Sci. Rep..

[136] Du T., Zhu G., Wu X., Fang J., Zhou E.M. (2019). Biotinylated Single-Domain Antibody-Based Blocking ELISA for Detection of Antibodies against Swine Influenza Virus. Int. J. Nanomedicine.

[137] Wood J.M., Weir J.P. (2018). Standardisation of inactivated influenza vaccines-Learning from history. Influenza Other Respir. Viruses.

[138] Kuck L.R., Byrne-Nash R., Gillis J., Bueter K., Couzens L.K., Eichelberger M.C., Rowlen K.L. (2018). VaxArray for hemagglutinin and neuraminidase potency testing of influenza vaccines. Vaccine.

[139] Nilsson C.E., Abbas S., Bennemo M., Larsson A., Hämäläinen M.D., Frostell-Karlsson Å. (2010). A novel assay for influenza virus quantification using surface plasmon resonance. Vaccine.

[140] Khurana S., King L.R., Manischewitz J., Coyle E.M., Golding H. (2014). Novel antibody-independent receptor-binding SPR-based assay for rapid measurement of influenza vaccine potency. Vaccine.

[141] Pierce C.L., Williams T.L., Moura H., Pirkle J.L., Cox N.J., Stevens J., Donis R.O., Barr J.R. (2011). Quantification of immunoreactive viral influenza proteins by immunoaffinity capture and isotope-dilution liquid chromatography-tandem mass spectrometry. Anal Chem..

[142] Pierce C.L., Williams T.L., Santana W.I., Levine M., Chen L.M., Cooper H.C., Solano M.I., Woolfitt A.R., Marasco W.A., Fang H. (2017). Immunocapture isotope dilution mass spectrometry in response to a pandemic influenza threat. Vaccine.

